# ResTRANS3D hybrid framework for data-efficient 3D medical image segmentation

**DOI:** 10.1016/j.isci.2026.115328

**Published:** 2026-03-12

**Authors:** Yibo Sun, Weitong Chen

**Affiliations:** 1 Adelaide University, South Australia, SA 5005, Australia

**Keywords:** health sciences, medicine, health informatics

## Abstract

Deep learning has become an important tool for 3D medical image segmentation, where learning effective representations from limited labeled data remains essential for practical deployment. Here, we present ResTRANS3D, a data-efficient self-supervised hybrid framework that combines a 3D-ResNet encoder with a multi-scale Transformer through a residual interaction mechanism to jointly model local spatial structures and long-range contextual dependencies. A dynamic position learning module generates adaptive positional representations conditioned on multi-scale features, while selective self-attention reduces the computational cost of global attention. The model is pretrained using a dual self-supervised strategy that integrates contrastive learning and image reconstruction. Experiments on multiple public 3D medical image benchmarks show that ResTRANS3D supports effective downstream segmentation, particularly when labeled data are limited. These results highlight the potential of hybrid representation learning to improve data-efficient 3D medical image analysis.

## Highlights

• ResTRANS3D combines 3D-ResNet and Transformer for hybrid volumetric learning
• Self-supervised pretraining improves segmentation under limited labeled data
• Selective self-attention reduces computation while maintaining segmentation accuracy
• Cross-dataset experiments support robust transferability across anatomical domains

## Graphical abstract

**Figure:**
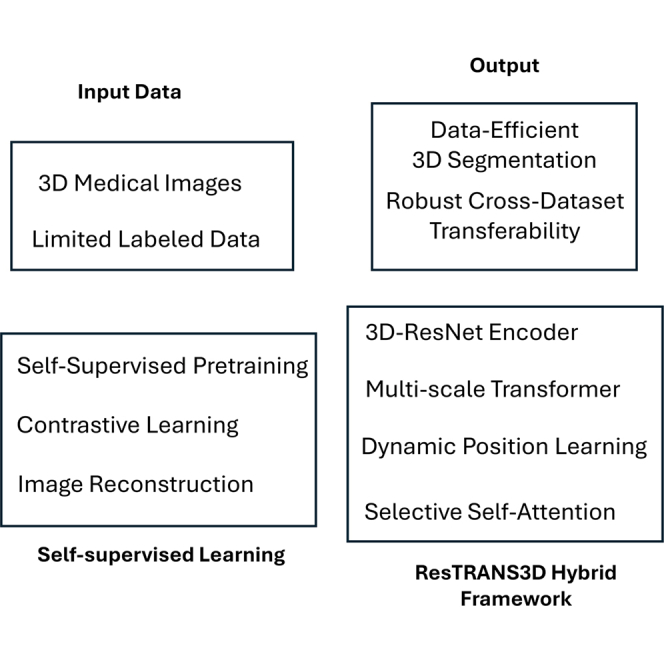

## Introduction

### Background

3D medical image segmentation is a fundamental task in computer-aided diagnosis (CAD) systems, playing an important role in clinical applications such as tumor localization, disease assessment, and surgical planning. In recent years, deep learning has achieved remarkable success in medical image analysis, significantly improving segmentation accuracy and robustness across various modalities and anatomical structures.^1^^,^^2^ Despite these advances, developing high-performance and clinically applicable 3D segmentation models remains a challenge. The goal of this work is to develop a data-efficient and computationally practical 3D medical image segmentation framework that can effectively leverage unlabeled data while balancing local and global representation learning.

Despite recent progress, 3D medical image segmentation faces three critical challenges. First, data scarcity remains a major bottleneck, as annotating volumetric medical images requires substantial expert effort and time.^3^^,^^4^ Second, feature representation imbalance persists, where existing models struggle to simultaneously capture fine-grained local details (e.g., lesion-bound areas) and long-range global contextual information (e.g., anatomical spatial relationships).^5^^,^^6^ Third, computational complexity poses a significant challenge, as the high dimensionality of 3D data makes many advanced models inefficient or difficult to deploy in practice.^7^^,^^8^

Current deep learning approaches attempt to address these challenges from different perspectives. Convolutional neural network (CNN)-based methods are effective in extracting local spatial features but exhibit limited capability in modeling long-range dependencies.^9^ Transformer-based approaches leverage self-attention mechanisms to capture global contextual relationships, yet often suffer from high computational cost and strong dependence on large-scale labeled data when applied to 3D medical images.^10^^,^^11^ Hybrid CNN-Transformer models balance global and local representations, and many existing methods adopt relatively rigid feature fusion strategies and predefined positional encoding schemes, which restrict their flexibility in modeling complex and scale-varying 3D anatomical structures.^12^^,^^13^

To mitigate annotation scarcity, self-supervised learning (SSL) has emerged as a promising paradigm by enabling representation learning from unlabeled data.^14^ SSL relieves reliance on manual annotations and improves feature transferability for downstream tasks by designing appropriate pretext tasks.^3^^,^^15^ In the context of 3D medical imaging, SSL requires substantial supervision to generalize effectively.^16^ However, existing SSL approaches for 3D medical image segmentation often focus on single pretext tasks or lack effective integration with global context modeling, limiting their robustness in data-scarce scenarios.^17^

Additionally, multi-modal medical image fusion is closely related to segmentation performance. Representative works include fusion frameworks based on co-occurrence filtering and local extrema in the NSST domain,^18^ content-aware GAN-based fusion methods,^19^ and joint-58-driven semantic-aware networks such as TSJNet.^20^ These studies highlight the value of multi-scale feature integration and semantic alignment. However, most of these approaches are limited to 2D imaging settings and do not incorporate self-supervised representation learning, leaving their extension to data-efficient 3D medical image segmentation insufficiently explored.

Although recent hybrid CNN-Transformer frameworks such as Swin UNETR^13^ and CoTr^7^ combine local convolutional features with global self-attention, most existing approaches treat the two components as parallel or loosely coupled feature extractors, relying on predefined or scale-agnostic positional encoding schemes. Such designs limit the adaptability of global context modeling to scale-varying anatomical structures and often show limited data efficiency when applied to large-scale 3D scenarios. Moreover, the high computational cost of dense or window-based self-attention remains a practical bottleneck for high-resolution volumetric segmentation.

To address these limitations in coupling strategy, positional modeling, and computational scalability, we propose ResTRANS3D, a data-efficient SSL framework for 3D medical image segmentation. ResTRANS3D integrates a 3D-ResNet encoder and Transformer branch to complement convolutional representations with global contextual cues rather than performing naive feature fusion. Instead of treating convolutional and Transformer modules as parallel feature extractors, ResTRANS3D constrains the Transformer branch to learn complementary global residual representations over convolutional features. This residual interaction design, combined with multi-task self-supervised pretraining, enables more transferable and robust 3D representations under limited annotated data. To further enhance spatial modeling, we introduce a dynamic position learning module (DPLM) that adaptively generates positional encodings conditioned on multi-scale volumetric features, overcoming the limitations of fixed or static positional embeddings.^21^

The main contributions of this work are summarized as follows:

(1) We propose ResTRANS3D, a data-efficient SSL framework for 3D medical image segmentation. Unlike existing CNN-Transformer hybrid models that mainly stack or fuse convolutional and attention blocks, ResTRANS3D introduces a residual interaction mechanism in which the Transformer branch serves as a global refinement module to complement convolutional representations. This design enables effective learning of transferable volumetric features under limited supervision.
(2) We introduce a DPLM that adaptively generates feature-conditioned and scale-aware positional representations from multi-scale 3D feature maps. Different from fixed sinusoidal encodings or static learnable embeddings used in prior works, DPLM adapts positional encoding to resolution changes and anatomical variability in volumetric data, improving spatial modeling for complex 3D medical structures.
(3) To address the high computational cost of global self-attention in 3D Transformers, we design a selective self-attention mechanism that restricts attention computation to a limited set of informative spatial locations. Compared with dense attention or existing sparse variants, the proposed mechanism significantly reduces computational complexity while preserving global contextual awareness, making Transformer-based modeling more practical for high-resolution 3D medical images.
(4) Extensive experiments on multiple public 3D medical image segmentation benchmarks (BraTS, Synapse, and ACDC) demonstrate that ResTRANS3D achieves competitive or superior segmentation performance compared with state-of-the-art methods such as nnFormer and UNETR, particularly under data-scarce conditions.

### Previous methods (related work)

#### SSL in 3D medical imaging

SSL has emerged as an efficient paradigm for representation learning, alleviating reliance on large-scale annotated data. In natural image cases, contrastive learning methods such as Sim- CLR^3^ and SwAV^5^ have shown promising results, along with generative methods like masked autoencoders (MAEs),^15^ which learn feature representations through image reconstruction. These cases have shown growing interest in transferring SSL to medical image analysis.

In the medical domain, SSL has been applied to tasks such as organ segmentation, lesion detection, and disease classification by leveraging unlabeled data through pretext tasks including rotation prediction, reconstruction,^12^ and contrastive objectives.^18^ Although most methods show the potential of SSL in reducing annotation dependency, most existing works have focused on 2D images or CNN-based architectures.^19^^,^^22^ The extension of SSL to 3D medical images combined with Transformer-based models^13^^,^^23^ remains challenging due to computational complexity and the difficulty of balancing local and global feature learning.

#### CNN-based segmentation methods

CNN-based architectures have long been the backbone of medical image segmentation. U-Net^1^ and its variants, such as nnU-Net,^2^ adopt an encoder-decoder structure with skip connections to effectively integrate low-level spatial details and high-level semantic features. nnU-Net further improves segmentation performance through adaptive configuration and task-specific optimization, achieving strong results across a wide range of medical datasets. In contrast, recent studies have explored incorporating SSL into CNN-based segmentation models. For example, BT-U-Net proposed by Punn et al.^24^ integrates the Barlow Twins self-supervision framework with U-Net, enhancing robustness under small-sample conditions. In addition, Models Genesis^16^ introduces self-supervised pretraining for 3D CNNs via image restoration, improving segmentation performance but still lacking global context modeling capability.

Despite the success of these cases, CNN-based segmentation methods contain inherent limitations. Due to the locality of convolution operations, these models struggle to capture long-range dependencies and global contextual relationships, which are crucial for accurately segmenting large or complex anatomical structures in 3D medical images.^11^ Moreover, most CNN-based approaches rely heavily on supervised learning and have limited ability to exploit unlabeled data, which restrict their scalability under data-scarce conditions.^16^

Recent studies have explored incorporating SSL into CNN-based frameworks to alleviate annotation dependency. For example, ResNet-based encoders have been pretrained using self-supervised objectives to improve downstream medical image segmentation performance.^14^^,^^23^^,^^25^ While such CNN-based SSL approaches enhance feature robustness, they still exhibit limited capacity for explicit global context modeling, particularly in 3D volumetric settings.

#### Transformer-based segmentation methods

Transformer-based architectures have attracted increasing attention in medical image segmentation due to their ability to model long-range dependencies via self-attention mechanisms. Vision Transformer (ViT)^10^ demonstrates that Transformer architectures can overcome the inductive bias limitations of convolutional networks when sufficient training data are available. This property makes Transformers particularly appealing for capturing global contextual relationships in medical images.

Several Transformer-based segmentation methods have been proposed in recent years. CoTr^7^ introduces Transformer modules to enhance global dependency modeling in medical image segmentation, while Swin Transformer-based approaches^13^ adopt hierarchical attention mechanisms to improve computational efficiency. MT-TransUNet^17^ further extends Transformer-based designs to multi-task medical image analysis, demonstrating the flexibility of attention-driven architectures in handling diverse segmentation and classification objectives.

Despite these advances, Transformer-based segmentation methods face notable challenges in 3D medical imaging. The quadratic complexity of self-attention leads to high computational and memory costs when processing volumetric data.^26^ Moreover, Transformer models are typically data hungry and exhibit limited robustness under small-sample or weakly supervised settings.^27^ These limitations restrict the direct applicability of pure Transformer-based approaches to large-scale, data-scarce 3D medical image segmentation tasks.

#### CNN-transformer hybrid methods

Hybrid CNN-Transformer models aim to combine the strong local feature extraction capability of convolutional networks with the global context modeling strength of Transformers. CoTr^7^ introduces Transformer modules into CNN-based architectures to enhance long-range dependency modeling but incurs high computational costs in 3D volumetric scenarios. Swin UNETR^13^ adopts hierarchical Transformer structures to improve efficiency; however, its reliance on fixed window partitioning and positional encoding limits adaptability to complex and scale-varying 3D anatomical structures. Similar hybrid designs demonstrate the potential of CNN-Transformer integration,^28^ yet often face challenges related to computational efficiency, positional modeling, and data dependency.

Recent studies indicate that SSL can effectively alleviate annotation scarcity in medical image analysis.^29^ Nevertheless, the integration of SSL with CNN-Transformer hybrid architectures for 3D medical image segmentation has received relatively limited attention, partly due to the compounded challenges of high-dimensional computation and the need to balance local and global feature representations.^11^ These challenges motivate the development of efficient hybrid frameworks that incorporate adaptive positional modeling and computationally efficient attention mechanisms within an SSL paradigm, as pursued in this work.

## Results

### Qualitative results

#### MRI segmentation results of brain tumors (BraTs)

**Figure 1:**
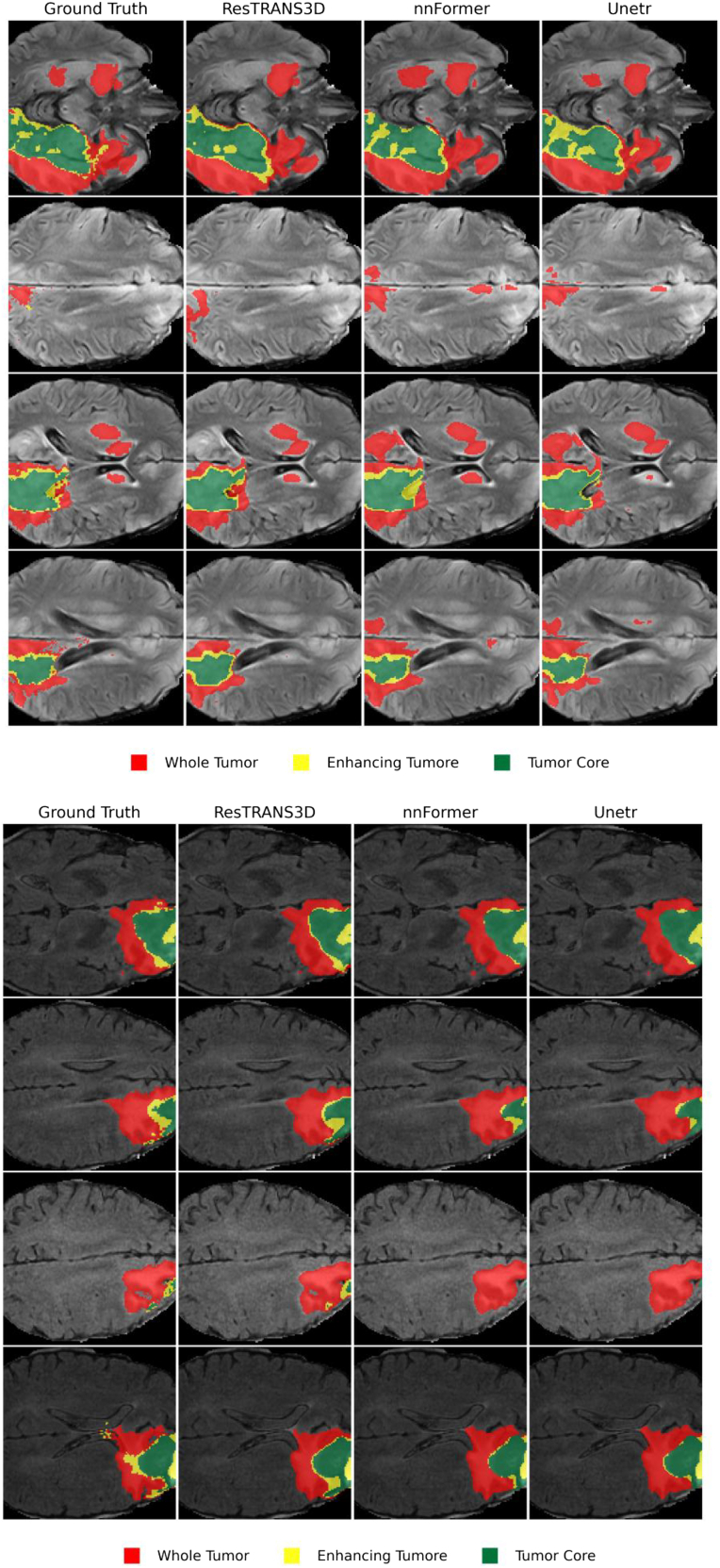
Qualitative segmentation results on the BraTS dataset Representative slices comparing ground truth and predictions from ResTRANS3D and competing models (3D-RMTNet, nnFormer, and UNETR). ResTRANS3D produces more accurate tumor boundary delineation and better preservation of small lesion structures, especially in challenging regions with heterogeneous intensity. Colors indicate different tumor subregions: red, whole tumor; yellow, enhancing tumor; green, tumor core.

We transferred the pretrained ResTRANS3D model to the BraTS brain tumor segmentation task and conducted comparative experiments with nnFormer^30^ and UNETR under the same experimental protocol. Representative qualitative segmentation results for “whole tumor,” “enhancing tumor,” and “tumor core” are shown in Figure 1. As illustrated in Figure 1, ResTRANS3D produced tumor segmentation results that are visually comparable to those of nnFormer across different tumor subregions, while maintaining coherent boundaries in most cases.

#### Multi-organ CT segmentation results (Synapse)

**Figure 2:**
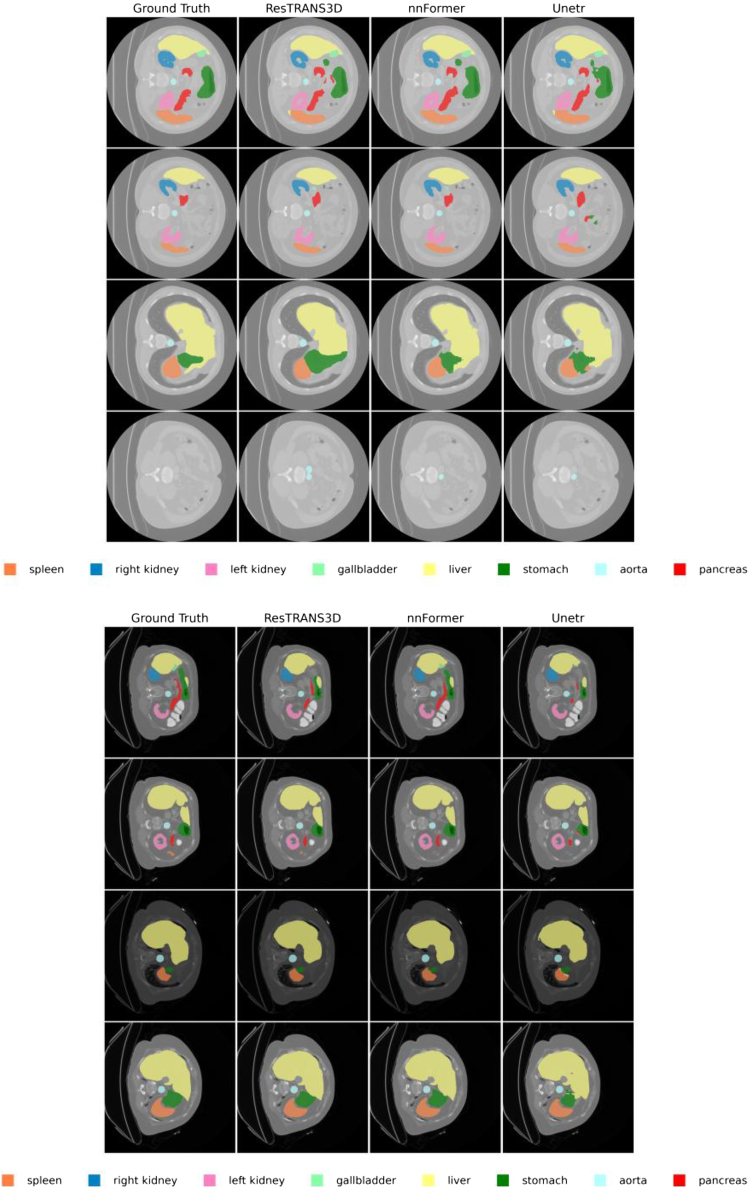
Visual segmentation results based on Synapse Visual comparisons between ground truth and model predictions demonstrate that ResTRANS3D achieves more precise segmentation of abdominal organs and improved boundary consistency. The model shows stronger robustness in regions with complex anatomical variations compared with baseline methods. Different colors represent different organs as indicated in the legend.

The dataset contains 3D abdominal CT scans with voxel-wise annotations for eight organs, including the spleen, left kidney, right kidney, gall bladder, liver, pancreas, aorta, and stomach. Following the same experimental protocol, we transferred the trained ResTRANS3D model to the Synapse multi-organ segmentation task and conducted comparative experiments against nnFormer and UNETR. Representative qualitative segmentation results for multiple organs are visualized in Figure 2, illustrating the comparative performance of the different methods.

#### Cine-MRI segmentation results of cardiac structure (ACDC)

**Figure 3:**
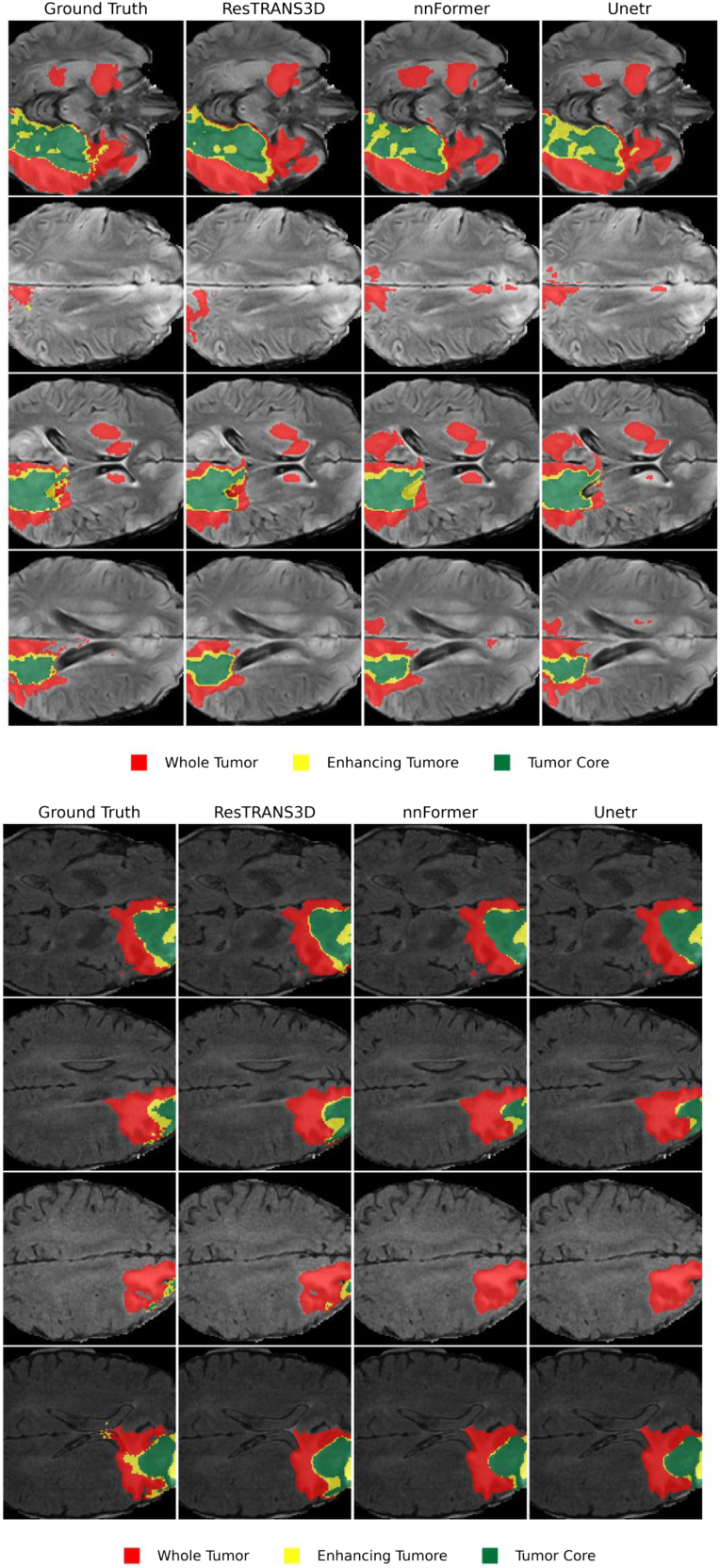
Qualitative segmentation results on the ACDC cardiac MRI dataset Comparison of segmentation outputs for left ventricle (LV), right ventricle (RV), and myocardium (Myo). ResTRANS3D provides more accurate structural delineation and better preservation of cardiac geometry across slices, highlighting its capability in modeling complex anatomical structures.

We transferred the pretrained ResTRANS3D model to the ACDC cine-MRI segmentation task and conducted comparative experiments against nnFormer and UNETR under the same experimental protocol. Representative qualitative segmentation results for left ventricle (LV), right ventricle (RV), and myocardium (Myo) are visualized in Figure 3.

As shown in Figure 3, ResTRANS3D produced segmentation results that are visually comparable to those of nnFormer across the three cardiac structures, while maintaining clear boundary delineation in most regions. Nevertheless, degraded performance can be observed in the frames affected by motion artifacts, such as blurred RV boundaries, which remain challenging cases for the current approach.

### Quantitative results

**Figure 4:**
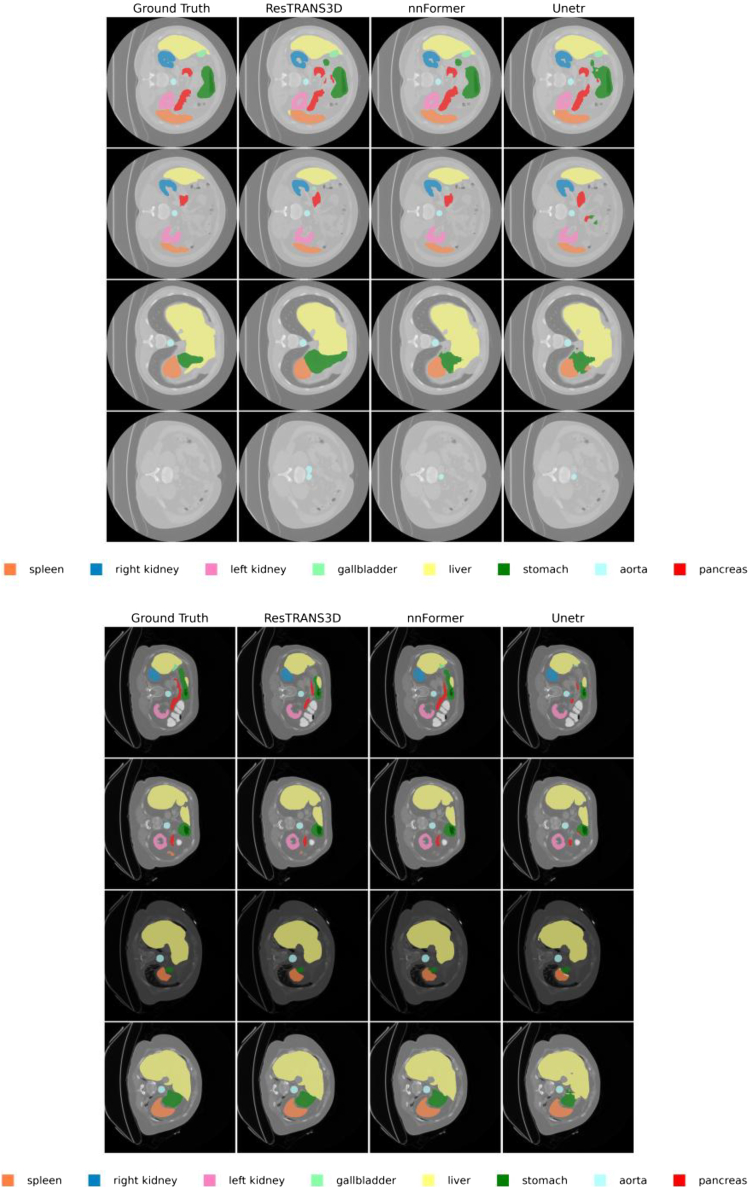
Average Dice score in BraTS Quantitative performance comparison of Re-sTRANS3D, nnFormer, and UNETR on three public benchmarks (BraTS, Synapse, and ACDC) showed that ResTRANS3D achieves competitive segmentation accuracy across datasets, indicating robust cross-domain generalization. Dice (%) represents the volumetric overlap between predictions and ground truth.

In our experiment, we used the Dice score to quantitatively assess the segmentation performance of different models across multiple anatomical structures. In this study, we evaluated the segmentation accuracy of ResTRANS3D, nnFormer, and UNet on the BraTS, Synapse, and ACDC datasets. Specifically, we report the average Dice scores of each model across the three datasets, as well as the Dice scores for each target region within each dataset. The quantitative results are summarized from Tables 1, 2, 3, and 4, and the overall comparison across datasets is illustrated in Figure 4.

**Table 1:** Average Dice score

Model	BraTS	Synapse	ACDC
ResTRANS3D	83.4%	84.5%	90.4%
nnFormer	86.3%	87.2%	92.3%
UNETR	72.1%	79.8%	87.5%

**Table 2:** Dice score of each tumor area in BraTS

Model	Whole tumor	Enhancing tumor	Tumor core
ResTRANS3D	90.1%	76.9%	83.2%
nnFormer	90.8%	80.9%	87.2%
UNETR	80.2%	57.8%	78.3%

**Table 3:** Dice score of each organ region in Synapse

Model	Spleen	Right kidney	Left kidney	Gall bladder	Liver	Stomach	Aorta	Pancreas
ResTRANS3D	88.2%	87.7%	86.8%	70.4%	95.8%	80.2%	91.4%	75.5%
nnFormer	91.1%	87.9%	86.6%	75.1%	96.4%	85.5%	92.1%	82.9%
UNETR	85.9%	85.3%	84.8%	63.4%	94.7%	75.6%	88.3%	60.4%

**Table 4:** Dice score of each structural region of ACDC

Model	LV	RV	Myo
ResTRANS3D	90.1%	88.5%	92.6%
nnFormer	91.7%	90.3%	94.9%
UNETR	84.5%	87.1%	90.9%

**Figure 5:**
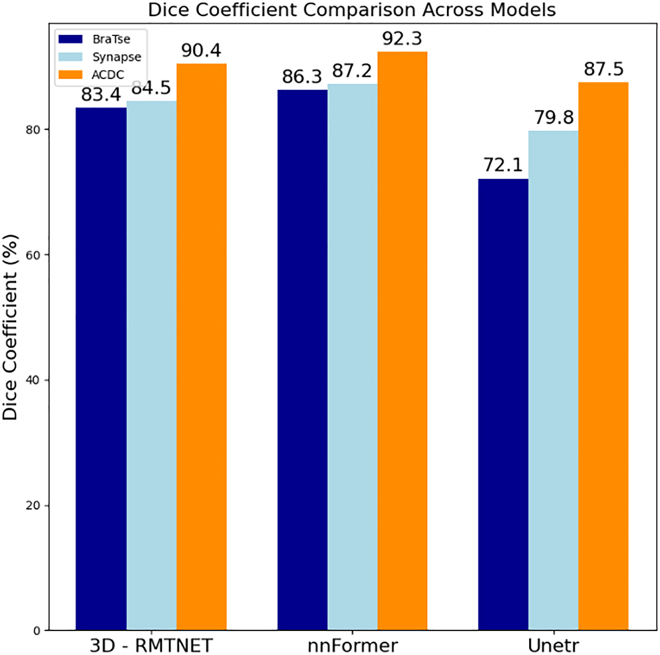
Dice scores of each tumor area in BraTS Performance comparison across different models on the BraTS dataset, measured by Dice scores. ResTRANS3D demonstrates improved segmentation accuracy across all tumor subregions, particularly in complex heterogeneous regions, suggesting better modeling of intratumoral variability.

In the BraTS dataset, ResTRANS3D achieved an average Dice score of 83.4%, demonstrating competitive performance compared with UNETR (Table 1). The Dice scores of each tumor region are shown in Figure 5. Under data-scarce conditions, self-supervised pretraining further enhanced model robustness. When only 10% of the labeled data were used, ResTRANS3D achieved a Dice score improvement of 5.2% (Table 10), which indicates its effectiveness under limited annotation.

**Figure 6:**
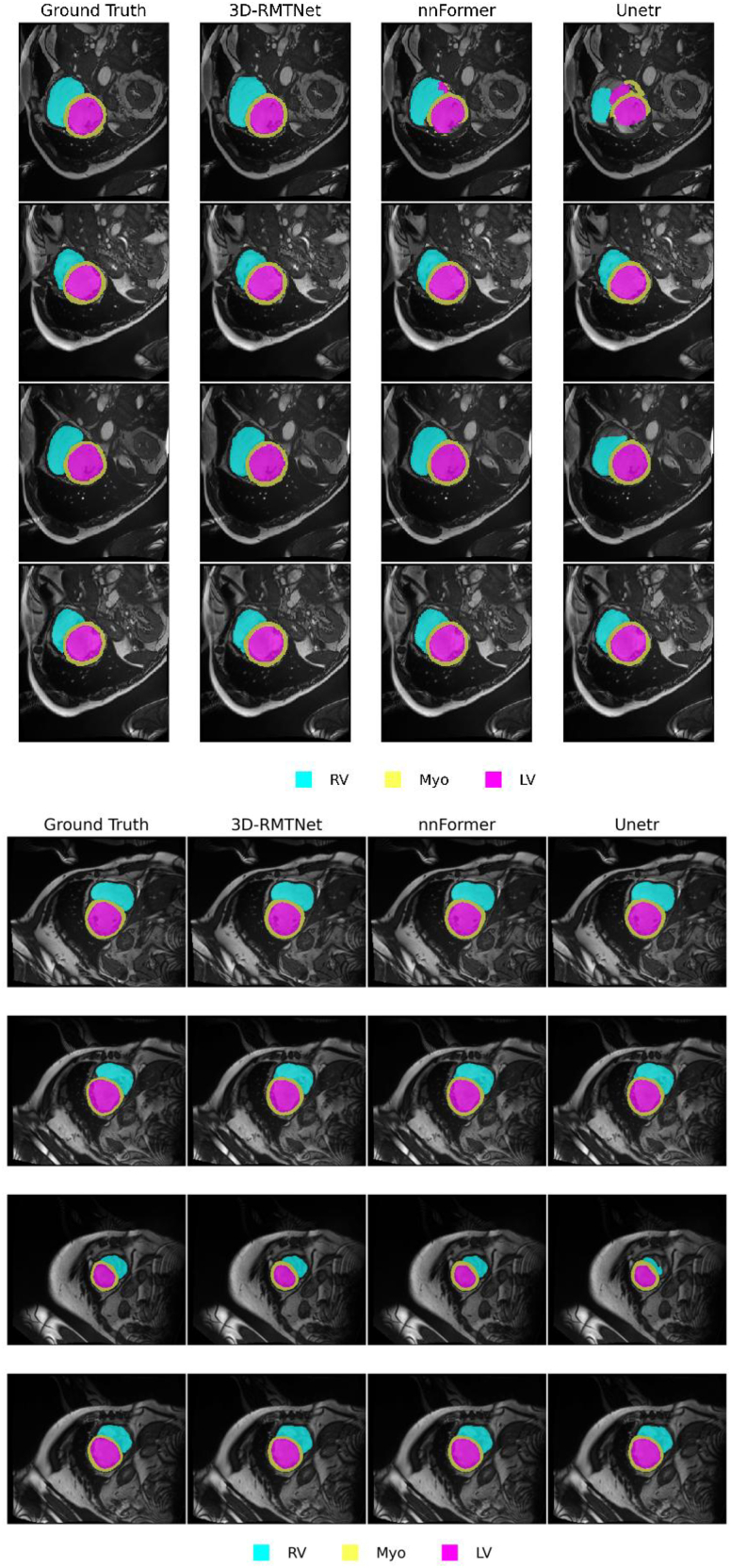
Dice scores of each organ region in Synapse Quantitative results comparing the segmentation performance of ResTRANS3D with competing models across multiple abdominal organs. ResTRANS3D consistently achieves higher or comparable Dice scores across most organ structures, indicating improved multi-organ representation capability and stable performance across anatomical regions. Dice (%) measures the volumetric overlap between predicted and ground-truth segmentations.

In the Synapse dataset, ResTRANS3D performed consistently across different organ segmentation tasks (Table 3). The Dice scores of each organ region are illustrated in Figure 6. High 219 Dice scores were achieved on larger organs such as the spleen and liver, while competitive performance was also observed on smaller and more challenging organs (e.g., pancreas and gall bladder) with complex shapes and ambiguous boundaries. This indicates that the proposed multi-scale Transformer encoder effectively captures long-range contextual information to support accurate organ delineation.

**Figure 7:**
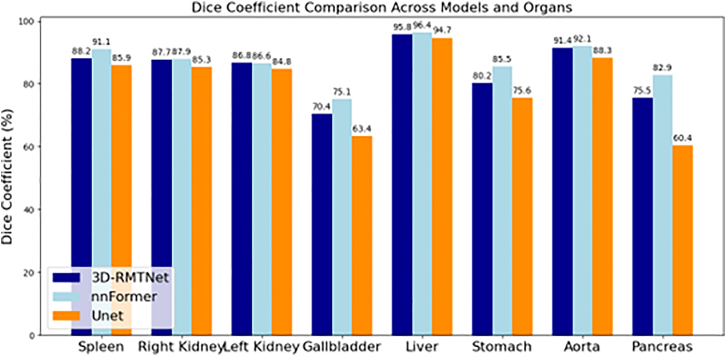
Dice scores of each structural region of ACDC Quantitative comparison across multiple abdominal organs in the Synapse dataset. ResTRANS3D achieves competitive performance across different anatomical structures, demonstrating strong capability in capturing organ variations and segmentation details.

In the ACDC dataset, ResTRANS3D achieved performance comparable to that of nnFormer in 225 LV, RV, and Myo segmentation (90.4% ± 0.8% vs. 92.3% ± 0.7%, 226 *p* > 0.05; Table 5). The Dice scores of each cardiac structure are presented in Figure 7. However, reduced robustness was observed in cases affected by motion artifacts, such as blurred RV boundaries caused by respiration, indicating that such scenarios remain challenging for the current approach.

**Table 5:** 5-Fold cross-validated average Dice scores

Model	BraTS (mean ± std)	Synapse (mean ± std)	ACDC (mean ± std)
ResTRANS3D	83.4% ± 1.2%	84.5% ± 1.5%	90.4% ± 0.8%
nnFormer	86.3% ± 0.9%	87.2% ± 1.1%	92.3% ± 0.7%
UNETR	72.1% ± 2.3%	79.8% ± 1.8%	87.5% ± 1.2%

#### Cross-validation

To improve the reliability and robustness of the reported results, we conducted 5-fold cross-validation on all datasets. Specifically, each dataset was randomly split into 5-folds at the subject level, where 4-folds were used for training and 1-fold was used for validation in each round. The reported performance was obtained by averaging the Dice scores across 5-folds and is summarized in Table 5.

To evaluate the computational efficiency of ResTRANS3D, we compared its complexity with nnFormer and UNETR in terms of FLOPs (floating-point operations), inference time per 3D volume, and GPU memory consumption. All measurements were conducted under the same hardware configuration and input resolution to ensure fair comparison. The results are reported in Table 6.

**Table 6:** Computational complexity comparison

Model	FLOPs (G)	Inference time (s/volume)	GPU memory (GB)
ResTRANS3D	18.6	1.2	8.3
nnFormer	22.3	1.8	10.5
UNETR	25.7	2.1	12.1

#### Cross-dataset generalization test

To evaluate the transferability of ResTRANS3D across anatomical domains, we first conducted single-source cross-dataset testing by pretraining on BraTS and fine-tuning/evaluating on Synapse and ACDC. BraTS was selected as the initial pretraining source because it provides diverse volumetric features (multi-modal MRI) and complex tumor subregions, which help the model learn more generalizable 3D representations.

To further verify consistent transferability, we additionally performed reciprocal cross-dataset generalization tests, including pretraining on Synapse (abdominal CT) and fine-tuning on BraTS or ACDC, as well as pretraining on ACDC (cardiac cine-MRI) and fine-tuning on BraTS/Synapse. All pretraining and fine-tuning settings were kept the same to ensure fairness. The results of both single-source and reciprocal transfers are reported in Table 7.

**Table 7:** Cross-dataset generalization performance (Dice, %)

Pre-train → fine-tune	Dice (%)
BraTS → Synapse BraTS → ACDC	82.7 89.1
Synapse → BraTS Synapse → ACDC	79.5 87.3
ACDC → BraTS ACDC → Synapse	81.2 80.4

Overall, ResTRANS3D maintained competitive performance under diverse cross-domain transfer settings, indicating that the proposed framework learns transferable volumetric representations that generalize well across datasets with different anatomies and imaging modalities.

#### Statistical significance test

To verify whether the performance improvements of ResTRANS3D over baseline models are statistically significant rather than caused by random variation, we perform paired *t* tests on Dice scores obtained from 5-fold cross-validation. The significance level was set to α = 0.05. The statistical results are summarized in Table 8.

**Table 8:** Paired *t* test results between ResTRANS3D and competing methods

Comparison pair	BraTS (*t*/*p*)	Synapse (*t*/*p*)	ACDC (*t*/*p*)
ResTRANS3D vs. UNETR	**4.23/<0.01**	**3.87/<0.01**	**3.52/<0.01**
ResTRANS3D vs. nnFormer	2.15/0.08	1.92/0.11	1.89/0.12

Bold values indicate statistically significant differences between methods (p < 0.05).

#### Computational complexity analysis

To evaluate the computational efficiency of ResTRANS3D independently from segmentation accuracy, we compared its complexity with nnFormer and UNETR in terms of the number of parameters, GFLOPs, and inference time per 3D volume. All measurements were conducted under the same hardware configuration, input resolution, and batch size to ensure a fair comparison. Notably, these efficiency metrics were measured using a single trained model without cross-validation, as computational complexity is determined by the model architecture, rather than data partitioning. The results are summarized in Table 9. As shown in Table 9, ResTRANS3D consistently exhibited lower computational cost across all metrics. Compared with the hybrid baseline nnFormer, ResTRANS3D reduced the number of parameters by approximately 47.8%, required about 75.2% fewer GFLOPs, and achieved a 41.9% reduction in inference time. Even when compared with UNETR, ResTRANS3D showed improved efficiency, with approximately 15.0% fewer parameters, 30.2% fewer GFLOPs, and 21.7% faster inference.

**Table 9:** Computational complexity comparison among segmentation models

Model	Params (M)	GFLOPs	Inference time (s)
ResTRANS3D	**78.6**	**52.9**	**1.8**
nnFormer	150.5	213.4	3.1
UNETR	92.5	75.8	2.3

Bold values indicate the best performance (e.g., highest Dice score or lowest computational cost) in each column.

These results indicate that the proposed selective self-attention mechanism effectively reduces computational overhead while maintaining competitive segmentation performance.

#### Limited label experiment

To evaluate the effectiveness of SSL under data-scarce conditions, we trained ResTRANS3D using only 10%, 20%, and 50% of the labeled data from the BraTS dataset. As a baseline, we adopted the same ResTRANS3D architecture trained from scratch without SSL pre-training on the corresponding labeled subsets, ensuring a fair comparison. As reported in Table 10, SSL pre-training consistently improved segmentation performance across all label ratios. In particular, when only 10% of the labeled data were available, the Dice score increased from 73.3% to 78.5%, corresponding to an absolute improvement of 5.2%. The performance gain gradually decreased as the proportion of labeled data increased, indicating that the proposed self-supervised strategy is especially effective under severe label scarcity.

**Table 10:** Dice performance under limited label settings on the BraTS dataset

Labeled data	With SSL	Without SSL	Improvement
10%	**78.5**	**73.3**	**+5.2**
20%	81.2	77.1	+4.1
50%	83.2	80.9	+2.3

Bold values indicate the best performance (e.g., highest Dice score or lowest computational cost) in each column.

### Ablation study

#### Component ablation experiments

To quantify the contribution of each core component in ResTRANS3D, including SSL, DPLM, and the selective self-attention mechanism, we conducted ablation experiments on the BraTS dataset. The results are shown in Table 14.

Removing the self-supervised pretraining led to a notable performance drop (−5.3% Dice), highlighting the importance of SSL in learning robust and transferable representations under limited annotation. Replacing the proposed DPLM with fixed sinusoidal positional encoding resulted in a 3.7% decrease in Dice score, while completely removing positional injection caused further degradation of performance (−3.1%), indicating that adaptive position learning plays a critical role in capturing spatial relationships in 3D volumetric data.

In addition, removing the selective self-attention mechanism and reverting to dense self-attention caused a substantial performance degradation (−13.2% Dice score). This result suggests that selective self-attention not only reduces computational complexity but also improves optimization stability and representation quality in high-dimensional 3D feature spaces. Replacing the proposed DPLM with fixed sinusoidal positional encoding led to a noticeable performance degradation, while completely removing positional injection further degraded accuracy, indicating that adaptive position learning plays a critical role in capturing spatial relationships in 3D volumetric data.

#### Parameter sensitivity analysis

To further investigate the robustness of the proposed model, we additionally conducted a parameter sensitivity analysis on three core hyperparameters on the BraTS dataset: (1) the number of sampled key points K in the selective self-attention module, (2) the weighting coefficient α_l_ used in DPLM, and (3) the temperature parameter τ in the contrastive learning objective. For each factor, we varied one parameter within a reasonable range while keeping all other settings fixed. Performance was evaluated using the Dice score, and efficiency is reported as GFLOPs. The results are summarized in Tables 11, 12, and 13.

**Table 11:** Sensitivity analysis on the sampling ratio *K* in selective self-attention (BraTS)

*K* (% of tokens)	5%	10%	20%	100% (full)
Dice (%)	82.1	83.4	83.6	83.7
GFLOPs	∼48.1	∼52.9	∼62.3	∼213.4

**Table 12:** Sensitivity analysis on the DPLM weight α_l_ (BraTS)

*α*_*l*_	0.05	0.1	0.2	0.5
Dice (%)	81.7	83.4	82.9	78.5
Δ (%)	−1.7	0.0	−0.5	−4.9

**Table 13:** Sensitivity analysis on the contrastive learning temperature *τ* (BraTS)

*τ*	0.05	0.1	0.2	0.5
Dice (%)	82.6	83.4	82.7	79.2
Δ (%)	−0.8	0.0	−0.7	−4.2

##### Sensitivity to key-point sampling ratio K

As shown in Table 11, increasing K improved segmentation accuracy but also increased the computational cost. In our experiments, setting K to 10% of tokens helped achieve a favorable balance between accuracy and efficiency, with performance close to full attention (K = 100%), while substantially reducing GFLOPs.

##### Sensitivity to DPLM weight α_l_

Table 12 indicates that ResTRANS3D is robust when α_l_ is within a moderate range (e.g., 0.05 ≤*α*_*l*_ ≤ 0.2). The best performance was achieved around α_l_ = 0.1. Excessively large α_l_ may overemphasize positional injection and suppress the original feature information, leading to a noticeable performance drop.

##### Sensitivity to contrastive temperature *τ*

As reported in Table 13, the model is relatively insensitive to *τ* in a reasonable interval (e.g., 0.05 ≤ *τ* ≤ 0.2), with performance changes within a small margin. When *τ* becomes too large, the similarity distribution between positive and negative pairs becomes less discriminative, resulting in segmentation accuracy degradation.

##### Summary

Overall, ResTRANS3D showed stable performance under moderate parameter variations, suggesting that the proposed design is not highly sensitive to these hyperparameters and is suitable for practical use.

## Discussion

### Ethical, regulatory, and translational considerations

Although SSL strategies reduce reliance on manual annotations, their application in medical image analysis raises important ethical, regulatory, and translational considerations. From an ethical perspective, SSL strategies typically rely on large volumes of unlabeled clinical data, which necessitates rigorous data governance, anonymization procedures, and strict compliance with institutional review board (IRB) protocols to mitigate potential privacy risks.

From a regulatory standpoint, models intended for clinical deployment are expected to demonstrate robustness, reproducibility, and transparency. Although ResTRANS3D was pretrained in a self-supervised manner, its downstream segmentation performance was systematically evaluated on standard public benchmarks, using established metrics, which aligns with current regulatory expectations in medical imaging research.

In terms of clinical translation, the data-efficient design of ResTRANS3D is highly relevant to real-world medical scenarios, where large-scale annotated 3D datasets are often scarce. By reducing annotation requirements while maintaining competitive segmentation accuracy, the proposed framework represents a practical step toward scalable AI-assisted 3D medical image analysis, particularly in resource-limited healthcare settings.

In general, we propose ResTRANS3D, a data-efficient SSL framework for 3D medical image segmentation, designed to alleviate the reliance on large-scale annotated datasets. By integrating a 3D-ResNet encoder with a multi-scale Transformer through a residual interaction mechanism, ResTRANS3D effectively captures both local spatial structures and long-range contextual dependencies in volumetric data. To enhance spatial representation, we introduce a DPLM that generates feature-aware positional encodings, enabling adaptive modeling of multi-scale anatomical structures. In addition, a selective self-attention mechanism is designed to significantly reduce the computational complexity of global attention while preserving global contextual information. Extensive experiments on public benchmarks demonstrate that ResTRANS3D achieves competitive segmentation performance across diverse anatomical structures, particularly under limited annotation. Overall, ResTRANS3D provides an efficient and scalable solution for 3D medical image segmentation in data-scarce scenarios, as well as a promising direction for integrating SSL with hybrid CNN-Transformer architectures in future medical image analysis applications.

## Discussion of results

### Performance analysis

In the BraTS dataset, the average Dice score of ResTRANS3D reached 83.4% 1.2% (Table 5), showing significant advantages over the UNETR model (72.1% 2.3%, *p* < 0.01, Table 8). Especially in the case of scarce data, SSL enhanced the robustness of the model: when only 10% of the labeled data were used, the segmentation performance was 5.2% higher than the model without SSL (Table 14), with the baseline being “ResTRANS3D without self-supervised pre-training” (Dice = 78.1%).

**Table 14:** Ablation experiment results on the BraTS dataset (Dice score, %)

Model variant	Dice (%)	Δ
ResTRANS3D (full model)	83.4	0.0
w/o SSL (supervised only)	78.1	−5.3
w/o DPLM (nopositional injection)	80.3	−3.1
Fixed sinusoidal PE (replace DPLM)	79.7	−3.7
w/o selective self-attention (dense attention)	70.2	−13.2

Δ indicates the performance drop relative to the full model.

In the Synapse dataset, ResTRANS3D performed well in segmenting large organs, such as the spleen (88.2%) and liver (95.8%). However, lower accuracy was observed for small organs with ambiguous boundaries, including the gall bladder (70.4%) and pancreas (75.5%), which can be attributed to the limited sampling of small anatomical regions by the selective self-attention mechanism.

In the ACDC dataset, ResTRANS3D achieved performance comparable to that of nnFormer in LV/RV/Myo segmentation (90.4% 0.8% vs. 92.3% 0.7%, *p* > 0.05, Table 8). Nevertheless, the model showed reduced robustness in the presence of motion artifacts (e.g., blurred RV boundaries caused by respiration), which indicated that these scenarios remain challenging for the current approach.

### Underperformance compared with nnFormer

Although ResTRANS3D achieved competitive overall segmentation performance, we observed that it underperforms nnFormer in certain small or structurally complex anatomical regions. Specifically, on the BraTS dataset, the Dice score for the “enhancing tumor” region was lower than that of nnFormer(76.9% vs. 80.9%, Table 2). A similar trend was observed on the Synapse dataset for pancreas segmentation (75.5% vs. 82.9%, Table 3), where the target organ is relatively small and exhibits ambiguous boundaries.

This performance gap can be attributed to architectural differences between the two models. nnFormer employs an interleaved Transformer design with dense attention, which facilitates refined modeling of fine-grained anatomical structures. In contrast, ResTRANS3D adopts a selective self-attention mechanism that focuses on a limited set of key sampling points to reduce computational complexity. While this design improves efficiency, it may miss critical cues in very small regions (e.g., lesions smaller than 5 mm), leading to reduced segmentation accuracy in these cases.

Despite this limitation, ResTRANS3D demonstrates markedly superior computational efficiency. Compared with nnFormer, ResTRANS3D uses only approximately 52.2% of the parameters, requires about 25% of the GFLOPs, and achieves a 41.9% reduction in inference time per 3D volume. Therefore, while sacrificing less than 4% Dice score in fine-grained regions, the proposed model provides a significantly lighter and faster solution, which makes it more suitable for resource-limited clinical workflows.

### Limitations of the study

Although the proposed ResTRANS3D framework demonstrates strong performance across multiple datasets and evaluation settings, several limitations remain.

First, the current SSL strategy adopts a fixed combination of contrastive learning and reconstruction objectives. More adaptive self-supervised schemes, such as dynamically weighted pretext tasks or masked volumetric modeling, may further improve representation transferability.

Second, although cross-dataset generalization experiments are conducted, the transfer settings are still limited to a small number of datasets and modalities. Future work will explore broader multi-source pretraining strategies across diverse anatomical regions and imaging protocols.

Third, while the selective self-attention mechanism improves computational efficiency compared with dense attention, further optimization through hardware-aware design or sparsity-driven attention may enable more efficient deployment in clinical environments.

Fourth, parameter sensitivity experiments indicate that the model is relatively robust within a reasonable adjustment range. However, automatic hyperparameter optimization strategies could further improve stability and usability in real-world applications.

Finally, this study mainly focuses on single-modality 3D medical image segmentation. Extending the framework to multi-modal and large-scale clinical datasets, as well as longitudinal imaging scenarios, remains an important direction for future research.

## Resource availability

### Lead contact

Further information and requests for resources should be directed to and will be fulfilled by the lead contact, Yibo Sun (yibo.sun@adelaide.edu.au).

### Materials availability

This study did not generate new unique biological materials.

### Data and code availability

• Data: The datasets used in this study (BraTS, Synapse, and ACDC) are publicly available from their official repositories.
• Code: The code supporting this study has been deposited at Zenodo and is publicly available at https://doi.org/10.5281/zenodo.18721206.
• Other: Any additional information required to reanalyze the data reported in this paper is available from the lead contact upon request.

## Acknowledgments

The authors thank all members of the school of Adelaide University. Grant numbers will be provided upon acceptance.

## Author contributions

Conceptualization, methodology, visualization, and writing – original draft, Y.S.; supervision, resources, formal analysis, project administration, and writing – review & editing, W.C. The authors have read and approved the final manuscript.

## Declaration of interests

The authors declare that they have no known competing financial interests or personal relationships that could have appeared to influence the work reported in this paper.

## STAR★Methods

### Key resources table

**Table:** 

REAGENT or RESOURCE	SOURCE	IDENTIFIER
**Deposited data**		

BraTS 2021 dataset	MICCAI BraTS Challenge	https://www.med.upenn.edu/cbica/brats2021/
Synapse multi-organ CT dataset	Synapse Repository	https://www.synapse.org/
ACDC cardiac MRI dataset	ACDC challenge	https://www.creatis.insa-lyon.fr/Challenge/acdc/
ResTRANS3D source code	This paper	https://doi.org/10.5281/zenodo.18721206

**Software and algorithms**		

PyTorch	PyTorch Team	https://pytorch.org/
MONAI	MONAI Consortium	https://github.com/Project-MONAI/MONAI
CUDA Toolkit	NVIDIA	https://developer.nvidia.com/cuda-zone

### Experimental model and study participant details

This study is based on publicly available medical imaging datasets. No new human or animal experiments were conducted. All datasets were collected and released by the original providers under their respective ethical approvals. We conducted experiments on three representative 3D medical image segmentation datasets:

(1) BraTS dataset for brain tumor MRI segmentation
(2) Synapse dataset for multi-organ abdominal CT segmentation
(3) ACDC dataset for cardiac Cine-MRI segmentation

### Method details

#### Datasets

The BraTS dataset^31^ is a widely used benchmark for brain tumor MRI segmentation. It consists of multi-modal MRI scans, including FLAIR, T1-weighted (T1w), contrast-enhanced T1-weighted (T1gd), and T2-weighted (T2w) images, collected from multiple institutions. The dataset provides voxel-wise annotations for three tumor subregions: edema (ED), enhancing tumor (ET), and non-enhancing tumor core (NET), making it particularly suitable for evaluating the ability of segmentation models to capture heterogeneous tumor structures.

The Synapse dataset^32^ is a widely used 3D CT benchmark for multi-organ segmentation and is commonly employed to evaluate the performance of medical image segmentation models in multi-class scenarios. The main challenge of this dataset lies in the large anatomical variability across different organs, which differ significantly in size, shape, and spatial distribution, thereby placing high demands on both local feature representation and global contextual modeling.

The ACDC dataset^33^ is a widely used benchmark for cardiac cine-MRI segmentation, consisting of annotated cardiac MRI scans from 100 patients. It is commonly employed to evaluate segmentation performance on key cardiac structures, including the left ventricle (LV), right ventricle (RV), and myocardium (Myo), which are clinically relevant for cardiac function analysis.

#### Model architecture

To address the shortcomings of traditional CNNs in capturing long-term dependencies, ResTRANS3D combines the advantages of 3D-ResNet and multi-scale Transformer models and learns rich feature representations from large-scale unlabeled 3D medical images through self-supervised learning. The proposed ResTRANS3D model consists of (1) a 3D-ResNet encoder composed of multiple residual stages for hierarchical local feature extraction; (2) a multi-scale Transformer encoder designed to model long-range contextual dependencies across volumetric features; and (3) a decoder built with 3D convolutional blocks and progressive upsampling layers with skip connections to recover spatial resolution for dense prediction. The model is first pretrained in a self-supervised manner on large-scale unlabeled 3D medical datasets to learn transferable volumetric representations, and subsequently fine-tuned end-to-end on labeled datasets for downstream segmentation tasks. The detailed architecture of each building block of ResTRANS3D is shown in Figure S1.

Unlike existing hybrid CNN–Transformer models that process convolutional and Transformer features in parallel, ResTRANS3D introduces a residual interaction mechanism that explicitly constrains the Transformer branch to learn complementary global residual representations over convolutional features. This design enforces tighter coupling between local and global representations, enabling more effective feature learning under limited supervision.

#### Feature extraction of ResTRANS3D

ResTRANS3D adopts a hybrid encoder to jointly capture local spatial details and global contextual information in 3D medical images. Specifically, a 3D-ResNet encoder is employed to extract hierarchical local features, while a Transformer module is introduced to model long-range dependencies across the entire volume.

Given an input 3D medical image

$$X\in R^{D\times H\times W}$$

the 3D-ResNet encoder produces multi-scale convolutional feature maps. The output feature map at the *l*-th layer is denoted as

$$F_{l}\in R^{C_{l}\times D_{l}\times H_{l}\times W_{l}}$$

where *C*_*l*_ represents the number of channels and *D*_*l*_×*H*_*l*_×*W*_*l*_ corresponds to the spatial resolution at that layer. These convolutional features provide strong local inductive bias and preserve fine-grained anatomical structures.

Based on the extracted convolutional representations, the Transformer module is subsequently applied to enhance global contextual modeling. Through the self-attention mechanism, the Transformer complements convolutional features by capturing long-range dependencies beyond local receptive fields, thereby improving global semantic consistency across the 3D volume.

#### Transformer-based global feature modeling

To model long-range dependencies beyond the local receptive fields of convolution, the Transformer module is introduced to process the high-level feature maps extracted by the 3D-ResNet encoder. The input feature maps are first reshaped into a sequence of feature tokens, each corresponding to a spatial region in the 3D volume.

Multi-head self-attention is then applied to dynamically capture global contextual relationships among these tokens. Unlike convolutional operations with fixed receptive fields, self-attention enables each token to adaptively attend to relevant regions across the entire volume, thereby enhancing global semantic representation. The Transformer outputs are subsequently reshaped back into volumetric form and forwarded to subsequent modules for further processing. The incorporation of adaptive positional modeling and computationally efficient attention mechanisms is described in the following subsections.

Multi-scale features extracted by the 3D-ResNet encoder are first enhanced by the Dynamic Position Learning Module (DPLM), which generates adaptive positional encodings for each scale. The resulting position-weighted representations are then processed by the selective self-attention mechanism, where attention is computed over a limited set of sampled key locations to reduce computational complexity. The refined features are subsequently fused and passed to the decoder for segmentation. As shown in Figure S2, multi-scale features are progressively processed by the proposed encoder and Transformer modules before being passed to the decoder for segmentation.

3D-ResNet-encoder The 3D-ResNet encoder is employed to extract hierarchical local features from the input 3D medical image. Given an input volume X ∈ RD×H×W, the encoder consists of multiple convolutional layers and residual blocks, which alleviate gradient vanishing and facilitate deep feature learning.

At the lth stage, the extracted feature map is defined as:

(Equation 1) $$F_{l}=\text{ResNet}\left(X;\Theta_{l}\right),l\in \left\{1,2,\ldots ,L\right\}$$

where $F_{l}\in {\left\{R\right\}}^{\left\{C_{l}\times D_{l}\times H_{l}\times W_{l}\right\}}$ defines the feature map extracted by the l-th layer, C_l_ denotes the number of channels, Θ_l_represents the learnable parameters of this layer, and D_l_×H_l_×W_l_ corresponds to the spatial resolution at scale l.

Since the Transformer processes sequential data, the 3D convolutional feature map needs to be flattened into a one-dimensional sequence, while positional information is incorporated to preserve spatial structure. Specifically, the feature map at the *l*-th layer is reshaped as *F* ^flat^ ∈ R^*Nl×Cl*^, where *N*_*l*_ = *D*_*l*_ × *H*_*l*_ × *W*_*l*_ denotes the total number of voxels in the feature map.

Multi-head self-attention is then applied to model long-range dependencies among these tokens. Unlike convolutional operations with fixed receptive fields, self-attention enables each token to adaptively attend to relevant regions across the entire 3D volume, thereby enhancing global contextual consistency. The resulting representations are reshaped back to volumetric form and forwarded to subsequent modules for further processing.

(1) DPLM learning module

Different from fixed sinusoidal positional encodings or window-based relative encodings used in existing hybrid frameworks, the proposed Dynamic Position Learning Module (DPLM) generates feature-conditioned positional representations from multi-scale volumetric features, allowing spatial encoding to adapt dynamically to varying anatomical scales and structures in 3D medical images.

Fixed positional encodings are often insufficient for modeling scale-varying and structurally complex anatomical patterns in 3D medical images. To address this limitation, we introduce a Dynamic Position Learning Module (DPLM) that generates adaptive positional representations conditioned on multi-scale feature maps.

For the l-th scale, the flattened feature representation, *F* ^flat^ R^*Nl×Cl*^ is used to dynamically predict positional encodings via a lightweight feed-forward network:

(Equation 2) $$P_{l}^{\text{flat}}={\text{MLP}}_{l}\left(F_{l}^{\text{flat}}\right),l\in \left\{1,2,\ldots ,L\right\}$$

where MLPl denotes a learnable fully connected network applied token-wise. The generated positional encoding P flat has the same dimensionality as *F* ^flat^.

(Equation 3) $$F_{l}^{\text{pos}}=F_{l}^{\text{flat}}+\alpha_{l}\cdot P_{l}^{\text{flat}}$$

where αl is a learnable scalar that controls the contribution of positional information at scale l. The spatial adaptive position design enables the model fully captures both local and global spatial relationships across different resolutions.

(2) Multi-scale Transformer

The position-enhanced features F pos are fed into a multi-scale Transformer encoder to capture long-range dependencies in the 3D volume. Each Transformer layer operates on a specific scale, enabling hierarchical global context modeling from coarse to fine resolutions.

Unlike convolutional operations with fixed receptive fields, self-attention allows each token to adaptively attend to informative regions across the entire volume, thereby enhancing global semantic consistency.

(3) Selective Self-attention Mechanism

By restricting attention computation to informative spatial locations rather than the entire 3D feature volume, the proposed selective self-attention mechanism explicitly addresses the computational scalability issue that remains insufficiently resolved in existing hybrid CNN–Transformer models.

Although self-attention is effective for global modeling, conventional dense self-attention incurs quadratic computational complexity $O\left(N^{2}\right)$. To improve efficiency, we introduce a selective self-attention mechanism that restricts attention computation to a limited set of informative key locations. The standard self-attention formulation is given by:

(Equation 4) $$Attention=Softmax\left(\frac{{\text{QK}}_{t}}{\sqrt{d_{head}}}\right)V$$

where Q, K, and V denote the query, key, and value matrices, respectively.

For a given query token q, attention is computed over K sampled key points across L scales:

(Equation 5) $${head}_{i}\left(q\right)=\sum_{i=1}^{L}\sum_{k=1}^{K}{\Lambda \left(\epsilon_{q}\right)}_{i}^{l,k}\Psi_{i}\left(F_{l}^{pos}\left({\overset{ˆ}{P}}_{l,q}+\Delta p_{i,l,k}\left(q\right)\right)\right)$$

where Λ_{l,k}(q) denotes the attention weight normalized by softmax over all sampled key points across scales.

$\hat{p}$_{l,q} is the reference 3D position of query token q at scale l, and Δp_{i,l,k}(q) is a learnable sampling offset.

F_lˆ{pos} denotes the positional feature map at scale l, which is sampled using differentiable trilinear interpolation.

Ψ_i is a learnable transformation (e.g., linear projection) applied to the sampled features.

By limiting attention computation to K sampled key points per scale, the computational complexity is reduced from O(Nˆ2) to O(NK).

(4) Multi-scale Feature Fusion

The outputs of multiple attention heads are concatenated and linearly projected to obtain the final encoded representation:

(Equation 6) $$F\_\text{deformable}=\text{Concat}\left({\text{head}}_{1}{,\text{head}}_{2},\ldots {\text{head}}_{H}\right)$$

(Equation 7) F_encoded=W·F_deformable

where H denotes the number of attention heads and W is a learnable linear projection matrix.

##### Transformer-decoder

The Transformer decoder progressively restores spatial resolution by upsampling and feature fusion. It consists of multiple 3D convolutional residual blocks and up-sampling layers. Skip connections are employed to fuse low-level features from the encoder with high-level Transformer features, preserving fine-grained details while incorporating global context. The decoder outputs a full-resolution feature map, which is subsequently mapped to the segmentation prediction.

#### Self-supervised learning tasks

To learn robust and transferable volumetric representations from unlabeled 3D medical images, we design a multi-task self-supervised learning strategy that provides complementary supervision from global, intermediate, and pixel-level perspectives. Specifically, contrastive learning enforces global representation consistency, auxiliary decoder-based prediction regularizes intermediate feature learning, and image reconstruction preserves fine-grained spatial and structural information.

(1) Contrastive Learning

For contrastive learning, two augmented views *x*_1_ and *x*_2_ are generated from the same input volume *x* through random spatial and intensity transformations. These two views form a positive pair, while views from other samples in the mini-batch are treated as negative pairs. Let *f*_1_ and *f*_2_ denote the feature representations of *x*_1_ and *x*_2_, respectively. This objective enforces global semantic consistency across different augmented views of the same 3D volume, encouraging the encoder to learn transformation-invariant and transferable volumetric representations.

The contrastive objective is optimized using an InfoNCE loss defined as:

(Equation 8) $$L_{\text{contrastive}}=-\log\frac{\exp\left(\text{sim}\left(f_{1},f_{2}\right)/\tau \right)}{\sum_{j=1}^{N}\exp\left(\text{sim}\left(f_{1},f_{j}\right)/\tau \right)}$$

In Formula 8, sim(·,·) denotes the cosine similarity, τ is a temperature parameter, and *N* is the number of samples in the mini-batch. This objective encourages feature representations of different views of the same volume to be close, while pushing apart representations of different volumes.

(2) Decoder-based auxiliary representation learning

To further regularize feature learning and encourage decoder consistency, the output feature map of the decoder is projected through a lightweight 1×1×1 convolutional layer to produce an auxiliary volumetric prediction:

(Equation 9) $$M=\text{C}\text{o}\text{n}\text{v}({\text{F}}_{\text{d}\text{e}\text{c}\text{o}\text{d}\text{e}\text{d}})$$

In Formula 9, *M*∈R^*C×D×H×W*^ denotes an auxiliary volumetric prediction generated during self-supervised pretraining, and *Conv* represents a 1×1×1 convolution. This auxiliary output is used to regularize representation learning rather than supervised segmentation. Unlike pixel-level reconstruction, this auxiliary prediction provides intermediate-level supervision, encouraging semantic consistency in decoder features and facilitating smoother transfer to downstream segmentation tasks.

(3) Image reconstruction

In addition, an image reconstruction task is employed to encourage the model to capture fine-grained structural information. A portion of the input volume is randomly masked or corrupted, and the network is trained to reconstruct the original image from the remaining visible regions. The reconstruction loss function is defined as:

(Equation 10) $$L_{reconstruction}={\left\|\hat{x}-x\right\|}^{2}$$

where *x* and $\hat{x}$ denote the original and reconstructed volumes, respectively. By preserving voxel-level appearance and structural information, the reconstruction objective complements contrastive learning by preventing excessive semantic abstraction.

By jointly optimizing contrastive learning, auxiliary decoder prediction, and reconstruction objectives, ResTRANS3D benefits from complementary supervision at multiple representation levels, enabling robust and transferable volumetric feature learning from unlabeled data for downstream 3D medical image segmentation.

#### Training Strategy and Optimization

To alleviate the scarcity of annotated 3D medical images, ResTRANS3D is first pretrained using the proposed self-supervised learning tasks described in previous subsection. During this stage, the network is trained on unlabeled 3D volumes to learn transferable volumetric representations without relying on manual annotations. The pretrained weights are used to initialize the convolutional encoder, Transformer modules, and interaction layers. After pretraining converges, the learned weights are transferred to initialize the downstream segmentation model.

##### Transfer learning and fine-tuning

After self-supervised pretraining, the entire network is fine-tuned end-to-end on labeled datasets for 3D medical image segmentation. No layers are frozen during fine-tuning unless otherwise specified. This transfer learning strategy enables ResTRANS3D to effectively leverage pretrained representations for downstream segmentation tasks, particularly under data-scarce conditions.

##### Segmentation loss and optimization

The segmentation model is optimized using a weighted combination of Dice loss and cross-entropy loss, and network parameters are updated via backpropagation.

(Equation 11) $$L_{\text{seg}}\lambda L_{\text{Dice}}+\left(1-\lambda \right)L_{\text{CE}},\lambda \in \left[0,1\right]$$

(Equation 12) $$L_{\text{Dice}}=1-\frac{1}{C}\sum_{c=1}^{C}\frac{2\sum_{i}p_{i,c}t_{i,c}+\epsilon }{\sum_{i}p_{i,c}+\sum_{i}t_{i,c}+\epsilon }$$

(Equation 13) $$L_{\text{CE}}=-\sum_{i}\sum_{c=1}^{C}t_{i,c}\;\log\left(p_{i,c}\right)$$

The segmentation model is optimized using a weighted combination of Dice loss and cross-entropy loss, as defined in (Equation 11), (Equation 12), (Equation 13). Dice loss addresses severe class imbalance at the region level, while cross-entropy loss enforces voxel-wise classification consistency. Network parameters are updated via backpropagation. The weighting factor λ is fixed to 0.5 in all experiments unless otherwise specified.

Where, *i* represents the voxel index in a 3D volume, and *C* defines the number of segmentation classes. $p_{i,c}$ represents the predicted probability of voxel *i* belongs to class *c* after softmax normalization, while $t_{i,c}$ defines the corresponding one-hot encoded ground truth label, and ϵ is a small constant added for numerical stability. The weighting factor λ balances region-level overlap optimization and voxel-wise classification accuracy and is fixed to 0.5 in all experiments unless otherwise stated. This loss design balances region-level overlap optimization and voxel-wise classification accuracy, and is consistently applied across all experiments.

#### Experimental setup

##### Experiment environment

To verify the effectiveness of ResTRANS3D in the task of medical image segmentation, we conducted a series of experiments on the datasets of brain tumor MRI segmentation (BraTs),^31^ multi-organ CT segmentation (Synapse),^32^ and cardiac structure Cine-MRI (ACDC).^33^ The experiment uses the same dataset and evaluation methods to ensure fair and consistent comparisons among all architectures. Hardware: Nvidia RTX 4060 GPU (16GB), Intel i9-13900K CPU, 64GB RAM.

Software: PyTorch 2.0, CUDA 12.1, OpenCV 4.8.0.

The experiments, including training, real-time enhancement and inference, were all conducted using PyTorch on a single Nvidia RTX 4060 GPU. Each model is trained using the same learning rate strategy, which includes the learning rate preheating stage. In this stage, we linearly increase the learning rate from 4e^−6^ to 4e^−4^ during the warm-up stage, followed by the PolyLR decay with a base learning rate of 3e^−5^. The AdamW optimizer is used for optimization.

The segmentation loss formulation is described in the Training Strategy and Optimization section, with λ fixed to 0.5. The batch size is set to 4, and all models are trained for 800 epochs.

#### Implementation details

All models were implemented using PyTorch 2.0 and trained on a single NVIDIA RTX 4060 GPU (16GB).

The AdamW optimizer was adopted with a warm-up learning rate schedule followed by polynomial decay.

Batch size was set to 4 and training was conducted for 800 epochs.

\item Synapse dataset for multi-organ abdominal CT segmentation

\item ACDC dataset for cardiac Cine-MRI segmentation

### Quantification and statistical analysis

#### Evaluation metrics

##### Dice similarity coefficient

The Dice Similarity Coefficient (DSC) is adopted as the primary quantitative metric to evaluate segmentation accuracy in this study, as it is widely used and well suited for class-imbalanced medical image segmentation tasks. The Dice score is defined as:

(Equation 14) $$\text{Dice}=\frac{2\times \left|A\cap B\right|}{\left|A\right|+\left|B\right|}$$

where A denotes the predicted segmentation region and B represents the ground-truth annotation.

##### Cross-validation protocol

To improve the robustness and reliability of the evaluation, a five-fold cross-validation strategy was adopted. Each dataset was split at the subject level into five mutually exclusive folds. In each run, four folds were used for training and one fold was used for validation, and the process was repeated until every fold had been used once as the validation set. The final performance was reported as the average across all five folds.

##### Statistical analysis

Statistical significance between competing methods was assessed using paired t-tests. A p-value less than 0.05 was considered statistically significant. All statistical comparisons were performed under the same experimental settings across datasets.

##### Computational complexity

Model efficiency was evaluated by the number of parameters, GFLOPs, and inference time. All efficiency comparisons were conducted under the same hardware setting to ensure fairness.
