# Supplementary material for: ResTRANS3D hybrid framework for data-efficient 3D medical image segmentation
Source: iScience. 2026 Mar 12;29(5):115328. doi: 10.1016/j.isci.2026.115328 (PMC13101297; doi:10.1016/j.isci.2026.115328)
Supplement: Document S1. Figures S1 and S2 [file mmc1.pdf]

iScience, Volume *29*

Supplemental information

ResTRANS3D hybrid framework for data-efficient 3D medical image segmentation

Yibo Sun and Weitong Chen

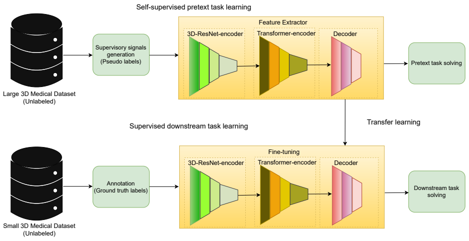

Figure S1: Overall architecture and training pipeline of ResTRANS3D. The framework consists of a 3D-ResNet encoder for hierarchical local feature extraction, a multi-scale Transformer encoder for modeling long-range contextual dependencies, and a convolutional decoder with skip connections for volumetric prediction. The model is first pretrained using a dual self-supervised strategy combining contrastive learning and image reconstruction, and then fine-tuned for downstream 3D medical image segmentation tasks. This design enables data-efficient representation learning from unlabeled volumetric data.

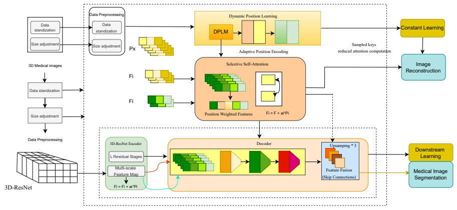

Figure S2: Module-level data flow of ResTRANS3D. The Dynamic Position Learning Module (DPLM) generates feature-conditioned positional encodings from multi-scale features, which are then integrated with convolutional representations through residual interaction. The resulting features are processed by the selective self-attention mechanism to capture global dependencies while reducing computational cost. Skip connections are used in the decoder to recover spatial details for final segmentation.
